# Development of a Reverse Line Blot Hybridization method for Detection of some Streptococcal/Lactococcal Species, the causative agents of Zoonotic Streptococosis/Lactococosis in farmed fish

**Published:** 2012-06

**Authors:** M Soltani, E Pirali, P Shayan, B Eckert, S Rouholahi, Shirazi N Sadr

**Affiliations:** 1 Department of aquatic animal health, faculty of veterinary medicine, university of Tehran, Tehran, Iran; 2 Department of parasitology, faculty of veterinary medicine, university of Tehran, Tehran, Iran; 3 Investigation institute of molecular biology and system transfer (MBST), Tehran, Iran

**Keywords:** Reverse line blot (RLB), *S. iniae*, *S. parauberis*, *S. agalactiae*, *L. garviae*

## Abstract

**Background and Objective:**

Streptococcosis/lactococcosis is the cause of high morbidity and mortality in aquaculture sector and to date a number of species of *Streptococcus* and *Lactococcus* genera including *S. iniae, S. agalactiae, S. dysagalactiae, S. parauberis, S. feacalis, L. garvieae* and *L. lactis* have been discriminated as the cause of disease in aquatic animals. Despite the use of diagnostic molecular methods for each of these bacterial species, no data is available on a suitable, rapid and simple simultaneous detection tool for these pathogens. This paper describes a simultaneous detection method which is PCR based on a reverse line blot (RLB) for rapid detection and differentiation of four species of genera *of Streptococcus* and *Lactococcus* genera consisting of *S. iniae, S. agalactiae, S. parauberis and L. garvieae* the most important agents of the disease in fish.

**Materials and Methods:**

A reverse line blot (RLB) assay was developed for the simultaneously identification of four species of *Streptococcus/lactococcus*consisting of *S. iniae, S. parauberis, S. agalactiae*and *Lactococcusgarvieae*. The assay employs one set of primer pair for specific amplification of the 16S rRNA gene. These were designed based on the nucleotide sequences of 16S rRNA gene sharing a homology region with *Streptococcus* spp. and *Lactococcus* spp. DNA was extracted from the pure bacterial colonies and amplified. A membrane was prepared with specific oligonucleotide for each bacterial species. PCR products were then hybridized to a membrane.

**Results and Conclusion:**

The amplification resulted in PCR product of 241 bp in length. No cross-reactions were observed between any of the tested bacterial species, and mixed DNAs from these four bacterial species were correctly identified. This RLB method is a suitable technique for a simultaneous detection of these species of bacterial fish pathogens that are some of the main causes of streptococcal/lactococcal infections in both freshwater and marine aquatic animals, and so we recommend its use for integrated epidemiological monitoring of streptococcosis/lactococcis in aquaculture industry.

## INTRODUCTION

At the present time, most of the common bacterial diseases are Gram-negative agents, however there are still some economically important Gram-positive pathogenic bacteria of fish, including some species of the genera of *Streptococcus* and *Lactococcus* (1, 2 3). The streptococcal/lactococcal infections usually cause high morbidity and mortality and last a long period of time in the farmed fish (2). To date, a number of species of *Streptococcus* and *Lactococcus*genera including *S. iniae, S. agalactiae, S. dysagalactiae, S. parauberis, S. feacalis, L. garvieae*and *L. lactis* have been discriminated as the cause of streptococcosis/lactococcosis in aquatic animals including fish (1, 2, 3, 4). Among these bacterial pathogens, *S. iniae* and *L. garvieae* have been reported as fish pathogens with high frequency while other species reported as low frequency (1, 3, 5).Despite many considerable reports on the outbreaks by these zoonotic pathogenic bacteria, minimum attempts have been allocated on their ecological distribution, the identification of their sources, and the modalities by which these bacterial pathogens are transmitted between fish (6, 7). Also, despite the existence of some diagnostic molecular methods for each of these bacterial species, no data available on a suitable, rapid and simple simultaneous detection tool for these pathogens (5, 8, 9, 10). The only available report is that by Mata et al. (2004) who developed a multiplex PCR method for detection of *S. iniae, L. garvieae*and *S. parauberis*. However, based on our work, this method is not reproducible according to the methodology described by these workers. This paper describes a simultaneous detection PCR based on a reverse line blot (RLB) for rapid detection and differentiation of four species of genera of *Streptococcus* and *Lactococcus* genera consisting of *S. iniae, S. agalactiae, S. parauberis* and *L. garvieae*, the most important agents of streptococcosis/lactococcosis in fish. This method can improve the rapid and simple detection for epizootiological purposes and preventive studies.

## MATERIALS AND METHODS

### Bacterial strains

*S. iniae* (accession no. FJ870987), *S. agalactiae* (type culture strain, department of microbiology, faculty of veterinary medicine, university of Tehran)*, S. parauberis (NCDO 2020)* and *L. garvieae* (accession no. HM055571) were used. All these bacterial species were isolated from the diseased fish except *S. agalactiae*.

### DNA extraction

DNA was extracted from the overnight cultures of the bacteria using DNA extraction kit for Gram positive bacteria (MBST, Tehran, Iran) and extracted DNA was stored at −20°C until the subsequent analysis. Briefly, 1.5 ml of the overnight bacterial culture was centrifuged for 10 min. at 8000×g. The pellet was resuspended in 100 µl bacterial homogenization buffer and centrifuged for 5 min at 8000×g. The pellet was resuspended in 150 µl hemogenization buffer and the bacteria as first lysed with 20 µl lysozyme (20 mg/ml) for at least 4 h at 37C. A volume of 180 µl lysis buffer was then added, mixed thoroughly and incubated for 10 min at 55°C. Twenty microliter proteinase K was added to the solution and the tubes containing the bacteria was incubated for 20 min at 55°C to degrade the proteins. A volume of 360 µl of binding buffer was then added before incubating for 10 minutes at 70°C. A volume of 270 µl ethanol (100%) was added to the solution and after vortexing, the complete volume was transferred to the MBST-column. MBST column was then first centrifuged and washed twice with 500 µl washing-buffer. Finally, DNA was eluted from the carrier with Elution buffer.

### PCR analysis

A pair of primers (P1 and P2) were designed based on the nucleotide sequences of 16S rRNA gene of *S. parauberis (NCDO 2020)* (Table 1). The DNA fragment between these two primers has a region with a variable nucleotide sequence between the corresponding nucleotide sequence in *S. agalactiae*, *S. parauberis*, *S. iniae* and *L. garvieae*. The 16S rRNA gene can be amplified partially using these two primers in these species of bacteria.

**Table 1:** Primers used for simultaneous detection of *S. agalactiae*, *S. parauberis*, *S. iniae* and *L. garvieae* using RLB assay.

Primer	Primer (5′→3′)	Modification	Accesstion no.	Target species
P1	GACGAACGCTGGCGGCGTGC	–	FJ009631	*Streptococcus-*sense all
P2	TACCTCACCAACTAGCTAAT	–	FJ009631	*Streptococcus*-antisense all
P3	TACCTCACCCAACTAGCTAAT	Biotin	FJ009631	*Streptococcus*-antisense all
P4	GGATAACTATTGGAAAC	C6-Aminolink	FJ009631	Catch all
P5	CTTGCACTAATCCAAA	C6-Aminolink	DQ193527	*S. iniae*
P6	TTTACACTAGACTGAT	C6-Aminolink	EU622516	*S. agalactiae*
P7	CTTGCTCTTTCCGGAT	C6-Aminolink	FJ405281	Unknown species
P8	CTTGCACTAGTCAGAT	C6-Aminolink	FJ009631	*S. parauberis*
P9	CTATTTTTATGAAGAGC	C6-Aminolink	AB267905	*L. garvieae*
P10	ACCGAGTGCTTGCACTCA	C6-Aminolink	JF156393	Uncultured bacterium clone ncd1809f10c1

The PCR was performed in a total reaction volume of 100 µl containing 10 µl of 10 × PCR buffer, 3 µl MgCl_2_ (50 mM), 2 µl of dNTP (10mM each), 0.5 µl Taq DNA polymerase (5 U/ µl, Fermentas), 2 µl of each primer (20 µM), 79/5 µl dH_2_O and 1µl of template DNA (approximately 100 ng). The reaction was repeated for 38 cycles under the following conditions: 5 min at 94°C, 45s at 94°C, 45s at 51°C, 45s at 72°C and a final extension step at 72°C for 10 min. Distilled water was used as negative control in each PCR reaction PCR products were separated on 1.8% agarose gel in 0.5× Tris-borate-ethylenediaminetetraacetic acid (EDTA) buffer and visualized using ethidium bromide and an UV illuminator. PCR products for reverse line blot (RLB) assay were prepared using primers P1 and P3.

### Reverse Line Blot (RLB) Assay

The procedure of RLB was described in the study published in detail by Gubbles et al. 1999 (11). A Biodyne C blotting membrane was activated in 16% EDAC solution at room temperature for 10 min. The membrane was shortly washed in double distilled water and placed in a mini-blotter (MBST / Iran). The oligonucleotide probes P4, P5, P6, P7, P8 and P9 at concentration of 1000 pmol in 250 µl 500 mM sodium hydrogen carbonate (pH 8.4) was transferred into the slots of mini-blotter and incubated for one min. After aspiration of solutions, the membrane was incubated for 10 min in 100 mMNaOH at room temperature. The membrane was then washed for 5 min using 2×SSPE (1 L 20×SSPE = 175.3 g NaCl, 27.6 g NaH_2_PO_4_, 9.4 g EDTA, pH 7.4), 0.1% SDS at 60°C and once at 42°C for 5 min. The membrane was then placed into the mini-blotter with slots perpendicular to the line pattern of applied probes. A volume of 45 µl of PCR products was diluted with 200 µl 2× SSPE, 0.1% SDS, heated to 95°C for 5 min and then cooled on ice. The slots were filled with diluted PCR product and hybridization was performed at 37°C for 60 min. After aspiration of the solution from the slots, the membrane was washed twice in preheated 2×SSPE, 0.1% SDS at 39°C under gentle shaking. The membrane was then incubated with 25 ml 2×SSPE, 0.1% SDS and 8 µl streptavidin-POD (Roch) for 30 min. at 39°C. The membrane was subsequently washed twice in preheated 2×SSPE, 0.1% SDS at 39°C and once at room temperature. Finally chemoluminescence detection was performed according to the standard procedure.

## RESULTS

**Fig. 1:**
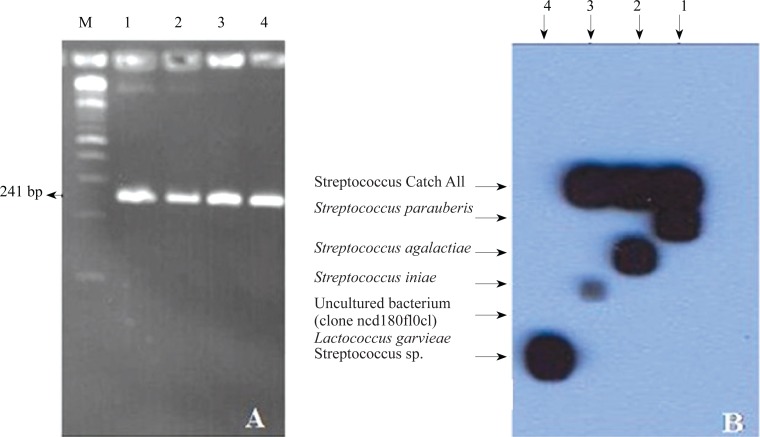
**A)** PCR products resulted from amplifying the DNAs extracted from *S. agalactiae*, S. *parauberis*, *L. garvieae* and *S. iniae* using primers P1 and P2. Lane 1 = *S. agalactiae*, Lane 2 = *S. parauberis*, Lane 3 = *L. garvieae* and Lane 4 = *S. iniae*, M = 100 bp DNA marker. **B)** PCR products were hybridized to the species specific oligonucleotides linked membrane followed by detection using chemoluminescence. Lane 1 = *S. parauberis*, Lane 2 = *S. agalactiae*, Lane 3 = *S. iniae* and Lane 4 = *Streptococcus* sp.

The extracted DNAs from colonies of *S. iniae, S. parauberis, S. agalactiae* and *L. garvieae* were amplified using primers P1 and P2 that are common primers able to amplify the genomic DNA of all *Streptococcus* spp. Fig. 1A shows that the primers amplified an expected PCR product of 241 bp in length. Interestingly, primers P1 and P2 flanked a variable region, its nucleotide sequence made us able to design oligo-nucleotides specific for each of tested bacterial species. To detect and discriminate the bacterial species from each other, species-specific oligo-nucleotides were designated from variable region (Table 1). To control the PCR products and hybridization procedure, additional primer (P4) from the homology region was also designed. The nucleotide sequence of P4 (catchall) is identical in 16S RNA gene of these four bacterial species, and was flanked through the primers P1 and P2 or P3. As negative control, two oligonucleotides were designed from the variable region of 16SRNA gene of unknown species of *Streptococcus* (P7) accession no. FJ405281 and of uncultured bacterium clone ncd 1809f10c1 (P10) accession no. JF156393 in GenBank. These primers were linked to the membrane using mini-blotter. The membrane was then hybridized with the PCR products resulted in amplifying of genomic DNA with primers P1 and P3. The hybridization results showed that all probes bound only to their respective target. Fig. 1B shows that all PCR products can react with the catch all oligonucleotide except *L. garvieae* and *Streptococcus* sp. registered under accession number FJ405281. The PCR products of all examined bacteria could not be annealed with the corresponding oligonucleotide derived from uncultured bacterium clone ncd1809f10c1 registered under accession number JF156393 in GenBank (Fig. 1B) and uncultured bacterium clone ncd1809f10c1 registered under accession number JF156393 in GenBank could not be annealed with the examined bacteria (Fig. 1B).

## DISCUSSION

Streptococcal/lactococcal infections became increasingly significant health problems in intensive aquaculture industry worldwide (12, 13). Reports by these fish pathogenic bacteria have been also increased in both invertebrates and humans (1, 3, 14, 15). The identification schemes for the causative agents, based on biochemical and antigenic features can barely differentiate these bacterial pathogens from other low virulent Gram-positive cocci such as *L. lactis* (1, 2). Studies involving the phenotypic characterization of the virulent species of these *Strepotococcus/Lactococcus*, collected from different fish species and countries, have been conducted using conventional methods and miniaturized systems and have given variable results (2, 10, 16). Therefore, more sensitive and time saving methods such as molecular techniques are required. In this work, we used a PCR based RLB method for a rapid simultaneous detection of four common fish pathogenic Gram-positive cocci belonging to genera of *Streptococcus* and *Lactococus*. The assay employed one set of primers for group-specific amplification of the 16S rRNA gene. No cross reaction was observed between any of the tested bacterial species. Also, the mixed DNAs from these four different bacterial species were correctly identified (Fig. 1A).The comparative nucleotide sequence analysis of 16S rRNA gene of some other bacterial species showed that the nucleotide sequence of sense- and antisense primers used in the present study had 100% homology to the corresponding sequence of *S. lactis* (accession no. M58837, *S. pyogenes* (accession no. AB002521), S. *dysagalactiae* subsp. *equisimilis* (accession no. EU075072) and *Entrococcus faecium* (accession no. FJ378708). This means that the 16S rRNA gene of these bacteria can be amplified with the used PCR primers. Interestingly from these bacteria, only the *S. dysagalactiae* subsp. *equisimilis* showed 100% homology with corresponding catch all sequence. *S. lactis*, *S. pyogenes*, *S. dysagalactiae* subsp. *equisimilis* and *E. faecium* showed differences in 3, 1 and 2 nucleotide(s) to the used catch all sequence, respectively. Also, the comparative nucleotide sequence analysis showed that the nucleotide sequence of antisense primer had 100% homology to the corresponding sequence of *Staphylococcus aureus* (accession no. Y15856), whereas the nucleotide sequence of its corresponding sequence to the sense primer had difference in only one nucleotide; and in catch all sequence they differed in 3 nucleotides. The comparative nucleotide sequence analysis also showed that the nucleotide sequence of sense primer and catch all oligo-nucleotide had 100% homology to the corresponding sequence of *S. equi* subsp. *zooepidemicus* (accession no. JN176353), whereas the nucleotide sequence of its corresponding sequence to the antisense primer had difference in two nucleotides. Furthermore, the sequence analysis showed that the corresponding nucleotide sequence of the used oligonucleotides (sense, antisense and catch all) in 16S rRNA gene in *E. coli* (accession nos. M25588, M24891, M24892 and M2489) had differences in 4, 9 and 3 nucleotides, respectively. Interestingly, the comparative sequence analysis showed that the used species specific oligonucleotides used in the present paper had no significant homology to the corresponding sequences of other above mentioned bacterial species.

In conclusion, the described method of PCR-RLB here in this work is a useful method for a rapid and simultaneous detection of some economically important streptococcal/lactococcal species in both aquatic and terrestrial animals. Use of such method can improve the integrated epidemiological monitoring of the disease outbreaks resulting in improving the protective measures such application of an effective vaccine. As vaccination of fish is now one of the useful protective tool to reduce the morbidity and mortality due to the disease outbreaks; this precise, simple and rapid simultaneous detection tool is helpful for improving of the preventive epidemiological studies particularly improving the protective measures such as production of an efficacious vaccine.
